# *Aspergillus* 6V4, a Strain Isolated from Manipueira, Produces High Amylases Levels by Using Wheat Bran as a Substrate

**DOI:** 10.1155/2014/725651

**Published:** 2014-03-02

**Authors:** Jessyca dos Reis Celestino, Ana Caroline Duarte, Cláudia Maria de Melo Silva, Hellen Holanda Sena, Maria do Perpétuo Socorro Borges Carriço Ferreira, Neila Hiraishi Mallmann, Natacha Pinheiro Costa Lima, Chanderlei de Castro Tavares, Rodrigo Otávio Silva de Souza, Érica Simplício Souza, João Vicente Braga Souza

**Affiliations:** ^1^Universidade Federal do Amazonas, UFAM, Faculdade de Ciências Farmacêuticas, Manaus, AM, Brazil; ^2^Instituto Nacional de Pesquisas da Amazônia, INPA, Laboratório de Microbiologia Médica, Manaus, AM, Brazil; ^3^Universidade Estadual do Amazonas, UEA, Escola Superior de Tecnologia, Manaus, AM, Brazil

## Abstract

The aim of this study was screening fungi strains, isolated from manipueira (a liquid subproduct obtained from the flour production of *Manihot esculenta*), for amylases production and investigating production of these enzymes by the strain Aspergillus 6V4. The fungi isolated from manipueira belonged to *Ascomycota* phylum. The strain *Aspergillus* 6V4 was the best amylase producer in the screening *assay of starch hydrolysis in petri dishes (ASHPD)* and in the *assay in submerged fermentation (ASbF)*. The strain *Aspergillus* 6V4 produced high amylase levels (335 UI/L) using wheat bran infusion as the exclusive substrate and the supplementation of this substrate with peptone decreased the production of this enzyme. The moisture content of 70% was the best condition for the production of *Aspergillus* 6V4 amylases (385 IU/g) in solid state fermentation (SSF).

## 1. Introduction

Amylases are enzymes with industrial importance. They have been used for the saccharification of starch in activities such as baking, fuel production, sugar production, and the textile and paper industries [1]. These enzymes are produced by most plants, animals, and microorganisms. However, the enzymes currently available are obtained through biotechnological bioprocesses utilizing microorganisms such as *Aspergillus* sp. and *Bacillus* sp. Some of these enzymes have special characteristics such as a tolerance to temperature and the bioprocesses from which they are produced are economically viable [2].

The literature describes innumerous amylases; however, the *α*-amylase, *β*-amylase, and glucoamylase are the most important economically. The *α*-amylase (1,4-*α*-glucan 4-glucanohydrolase, EC 3.2.1.1.) is an enzyme that breaks the connections *α* (1,4) from polysaccharides which have three or more units of D-glucose [3]. The attack occurs at various points of the chain simultaneously and the first hydrolysis products are always oligosaccharides of 5–7 glucose units. The *β*-amylase hydrolyzes the glycosidic linkages from the nonreducing end of the polysaccharides separating two glucose units and forming *β*-maltose [4]. The amyloglucosidase or glucoamylase (1,4) (1,6)-*α*-D-glucan glucanohydrolase, EC 3.2.1.3 breaks *α*-1.4 and *α*-1.6 bonds from the nonreducing end [3, 5].

In the Amazon rain forest the vegetal biomass is rapidly degraded by microbes; this is due to weather conditions, humidity, substrate abundance, and the potential of microorganisms in producing enzymes [6]. The cassava (*Manihot esculenta*) is an important source of food for the Amazon population and it is, as well as it's by-products, potential good substrates for searching amylolytic microorganisms. The aim of this study was screening fungi strains, isolated from manipueira (a liquid subproduct obtained from the flour production of *Manihot esculenta*), for amylases production and investigating production of these enzymes by the strain *Aspergillus* 6V4.

## 2. Materials and Methods

### 2.1. Fungi Isolation

Approximately 1 mL of manipueira (a liquid subproduct obtained from the flour production of *Manihot esculenta*) was subjected to successive dilutions (1 × 10^−1^–1 × 10^−5^) and 100 *μ*L aliquots was plated on a medium composed of 1.0% soluble starch, 0.25% peptone, and 0.25% yeast infusion. The plates were incubated at 35°C and monitored daily over 5 days for isolation of filamentous fungi. The isolated colonies were purified and stored in potato dextrose agar (PDA) at 7°C [7].

### 2.2. Screening 1:* Assay of Starch Hydrolysis in Petri Dishes (ASHPD)*

The radial growth and the halo of starch hydrolysis were observed during the growth of the strains in a culture medium that has starch as the main carbon source (1.0% soluble starch, 0.25% peptone, and 0.25% yeast infusion). The observation of hydrolysis halos was undertaken with a 1% iodine solution [8].

### 2.3. Screening 2:* Assay in Submerged Fermentation (ASbF)*

The strains were submitted to a submerged bioprocess in order to evaluate the production of extracellular amylases [7]. 20 mL of medium composed of 1.0% soluble starch, 0.25% peptone, and 0.25% yeast infusion was placed into 125 mL Erlenmeyer flasks; this was inoculated with 1 × 10^5^ cell/mL. The bioprocess was conducted at 35°C, with orbital agitation (150 rpm), for 72 hours. The fermented material was centrifuged at 10,000 g for 10 min and the supernatant was used for the determination of amylase activity.

### 2.4. Amylases Production Submerged Fermentation Using Wheat Bran as the Substrate and the Influence of Peptone Supplementation in the Enzyme Activity

The strain selected in the screening assays (Sections 2.2 and 2.3) was submitted to a submerged bioprocess using wheat bran infusion as the substrate. The wheat bran infusion was chosen as substrate since we believe that the hot water is able to extract the soluble starch from the wheat bran allowing the separation of this component leading to the amylases induction. 50 mL of wheat bran infusion (60 g of wheat bran was mixed with 1 L of water at 80°C and then filtered with gauze) was transferred to 125 mL Erlenmeyer flasks. A spore's suspension was prepared and the culture medium was inoculated with 1 × 10^4^ spores/ml. The Erlenmeyers were incubated in an orbital shaker (100 rpm) at room temperature for 96 hours. Samples were collected every 24 hours in order to evaluate the biomass production (dry weight) and the amylases production. The effect of the culture medium supplementation with peptone 5% (w/w) was investigated.

### 2.5. Amylase Production in Solid State Fermentation (SSF)

Wheat bran (15 g) was added to 250 mL Erlenmeyer flasks; this substrate was moisturized and inoculated with the strain selected in the screening assays (Sections 2.2 and 2.3), 1 × 10^4^ spores/g. The bioprocess was incubated at room temperature for 96 hours. These experimental conditions evaluated the influence of moisture contents of 50, 60, 70, and 80% in amylases production. The enzymes were extracted by adding 1g of the SSF to 10 mL water in an Erlenmeyer flask and incubating it in orbital agitation (100 rpm) for 30 minutes.

### 2.6. Determination of Amylase Activity

The reaction mixture consisted of 30 *μ*L enzymatic solution (culture filtrate) and 30 *μ*L substrate solutions (soluble starch in 0.2 M acetate buffer, pH 5.0). This mixture was incubated at 42°C for 30 minutes; this reaction was stopped by adding 200 *μ*L of 0.2 M HCl. The reaction then received 40 *μ*L of iodine solution (0.30% KI, 0.03% I_2_). The control was prepared according to the procedure described above but the enzyme was replaced by an equivalent volume of distilled water (control substrate). Another control was performed by replacing the starch solution per the same volume of acetate buffer control (enzyme). The absorbance was determined at 600 nm in a spectrophotometer. One unit of amylase activity was defined as the amount of enzyme required to hydrolyze 1.0 mg of starch per minute under the assay conditions [9].

## 3. Results

The cassava flour byproducts were not previously well studied as a source of amylase producers. In the fungal isolation a culture medium containing starch was used as the main carbon source. 20 cultures from mitosporic fungi were obtained; they belonged to *Ascomycota* phylum with most of them belonging to the genera *Aspergillus* and *Penicillium*.

In order to select a strain able to produce high levels of amylase, two different screening assays were carried out, *ASHPD* and *ASbF*. In the *ASHPD*, all isolates produced amylases and ten of them produced an enzymatic index greater than 1.5 (Table 1). In the *ASbF* the amylase activities ranged from 2.0 to 36.2 IU/L (Table 1). The strain *Aspergillus* 6V4 was selected for the next stages of this study for presenting one of the highest amylase production in *ASbF.*

**Figure 1:**
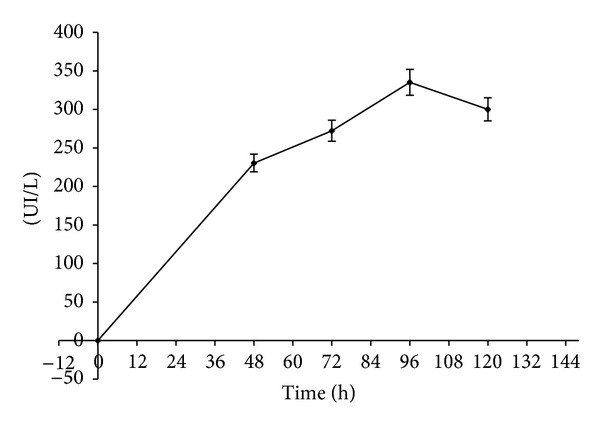
Production of amylase from *Aspergillus* 6V4 in SbF using the wheat bran infusion as substrate.

An infusion obtained from wheat bran was used as the substrate for amylase production; the infusion was obtained through infusion and a Sbf was carried out with the strain *Aspergillus* 6V4. The maximum enzymatic activity (335 U/L) was obtained at 96 h (Figure 1).

In order to investigate if wheat bran was an adequate substrate for amylase production, an experiment evaluating the influence of the supplementation with peptone was carried out. The supplementation of 5 g/kg resulted in the decreased the amylase activity in 14,3%.

**Figure 2:**
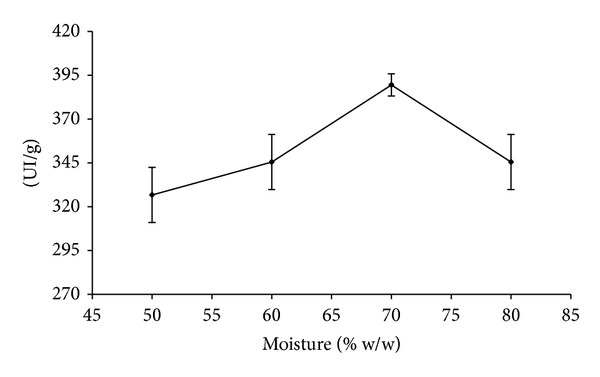
Production of *Aspergillus* amylases 6V4 in SSF using wheat bran as the substrate containing different moisture.

Whilst assessing the influence of the moisture in the production of amylase in SSF, it was observed that 70% moisture was the best condition (Figure 2).

## 4. Discussion

The microorganisms isolated in this study were from the *Ascomycota* phylum; such groupings are known as good amylases producers. The *Aspergillus* spp. has been used for production of amylases that are currently on the market; these genera produce over 200 extracellular enzymes and several of these have industrial importance [10, 11].

The index assay (assay plates) did not correlate with the results of amylase production in a liquid medium (regression *R* ^2^ = 0.09). This discrepancy can be explained since the study in agar plates has a different environmental condition from that carried out in the submerged bioprocess [12]. The screening assay showed amylases production from 2.0 to 36.2 IU/L. These enzyme concentrations are low; however, they were obtained from experiments that were not optimized. The strain *Aspergillus* 6V4 had the highest enzyme production and was selected for the remaining experiments.

The *Aspergillus* 6V4 colonies presented masses of yellow-green spores on the upper surface and reddish-gold on the lower surface. It's hyphal occurred by thread-like branching and produced mycelium. The hyphae were spetade and hyaline and it was observed conidiophores (colorless) and conidiaspores in phialides uniseriate and biseriate. This morphological information included this microorganism in the *Aspergillus flavus* group. The ITS region of his rDNA has been investigated in order to define specie.

Agroindustrial residues have been used in research bioprocesses as a substrate because they are relatively inexpensive and they have an appropriated content of carbon and nitrogen sources [9, 13, 14]. In the present work, a wheat bran infusion was used for producing amylases by SbF with *Aspergillus* 6V4. This strategy resulted in the increase of amylase production (335 IU/L) in 9.25 times. This amylase level is comparable to that described in other studies [15, 16] and this demonstrates that the wheat bran is a suitable substrate. The supplementation of wheat bran infusion with peptone decreased the production of amylases. This is probably a result of the effect of metabolic repression and demonstrates that the wheat bran infusion has nitrogen sources in the desirable amount for the enzyme production [9, 13, 14].

The solid state bioprocess has been evaluated for the production of enzymes which are of industrial interest. They allow the use of agroforestry residues and exhibit both higher productivity and lower operational costs [17]. Under the experimental conditions 385 IU/g was produced. Comparing SbF with SSF, the latter produced more enzyme in 1 g of solid state medium than what was observed in a 1 L of submerged fermentation medium. Similar results have previously been demonstrated by [17]. The moisture trial demonstrated that 70% w/w was the best for amylase production. This result agrees with previous studies that showed that moisture content of approximately 70% was the most suitable for the production of enzymes which are of industrial interest.

Optimized production and characterization of the enzymes produced by the microorganism isolated in this study are presented as an appropriate strategy to more adequately evaluate the biotechnological potential of this new source of amylases [14, 18]. This work demonstrated that cassava byproducts can be used as a source of amylase producers; the organisms isolated from these substrates belonged to *Ascomycota* phylum and the strain *Aspergillus* 6V4 produced high amylase levels and needs to be further investigated.

## Figures and Tables

**Table 1:** Results of amylase production by the isolated strains in the *assay of  starch hydrolysis in petri dishes (ASHPD)* and in the assay in *submerged fermentation (ASbF)*.

Isolates	ASHPD	ASbF
	Enzyme index (mm/mm)*	Amilase activity (IU/L)
*Penicillium* 1B10-5	1.90	28 ± 2
*Penicillium* 2B10-5	1.3	10 ± 4
*Aspergillus* 3V10-5	1.1	19 ± 9
*Fusarium* 4V10-5	1.1	25 ± 7
*Aspergillus* 5V10-5	1.1	10 ± 2
*Penicillium* 6V10-5	1.35	19 ± 1
*Penicillium *7V10-5	1.78	2.0 ± 0.3
*Aspergillus* 8V10-3	1.26	12 ± 3
*Paecilomyces* 9V10-5	1.75	12 ± 5
*Aspergillus* 10V10-5	1.74	32 ± 0.1
*Aspergillus* 1V-4	1.55	12.5 ± 0.4
*Aspergillus* 2V-5	1.39	7.4 ± 0.4
*Aspergillus* 3V-5	2.2	33 ± 4
*Aspergillus* 4V-5	1.90	2.4 ± 0.6
*Aspergillus* 5V-4	1.54	15 ± 9
*Aspergillus* 6V4	1.90	36.2 ± 2
*Penicillium* 7V-4	1.58	32.5 ± 0.3
*Penicillium* 8V-4	1.34	10 ± 5
*Aspergillus* 9V-5	1.29	11 ± 2
*Aspergillus* 10V-5	1.37	15 ± 3

*(Diameter of colony + Halo hydrolysis)/(diameter of colony).
